# Super-crystals in composite ferroelectrics

**DOI:** 10.1038/ncomms10674

**Published:** 2016-02-24

**Authors:** D. Pierangeli, M. Ferraro, F. Di Mei, G. Di Domenico, C. E. M. de Oliveira, A. J. Agranat, E. DelRe

**Affiliations:** 1 Dipartimento di Fisica, Università di Roma ‘La Sapienza', Rome 00185, Italy; 2 Center for Life Nano Science@Sapienza, Istituto Italiano di Tecnologia, Rome 00161, Italy; 3 Department of Applied Physics, Hebrew University of Jerusalem, Jerusalem 91904, Israel

## Abstract

As atoms and molecules condense to form solids, a crystalline state can emerge with its highly ordered geometry and subnanometric lattice constant. In some physical systems, such as ferroelectric perovskites, a perfect crystalline structure forms even when the condensing substances are non-stoichiometric. The resulting solids have compositional disorder and complex macroscopic properties, such as giant susceptibilities and non-ergodicity. Here, we observe the spontaneous formation of a cubic structure in composite ferroelectric potassium–lithium–tantalate–niobate with micrometric lattice constant, 10^4^ times larger than that of the underlying perovskite lattice. The 3D effect is observed in specifically designed samples in which the substitutional mixture varies periodically along one specific crystal axis. Laser propagation indicates a coherent polarization super-crystal that produces an optical X-ray diffractometry, an ordered mesoscopic state of matter with important implications for critical phenomena and applications in miniaturized 3D optical technologies.

## Abstract (web summary)

Crystalline materials are formed by the periodic order of atomic unit cells. Here, Pierangeli *et al*. report the formation of a ferrelectric super-crystal with micrometric unit cell from modulated nanoscale disorder in potassium-lithium-tantalate-niobate, which is potentially useful for engineering ordered states out of disorder.

## Graphical abstract

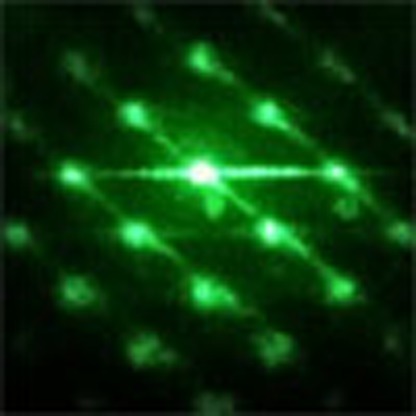

Textbook models of global symmetry-breaking include a low-symmetry low-temperature state with a fixed infinitely extended coherence. In contrast, the spontaneous polarization observed as spatial inversion symmetry is broken during a paraelectric–ferroelectric phase transition generally leads to a disordered mosaic of polar domains that permeate the finite samples^1^. Coherent and ordered ferroelectric states with remarkable properties of both fundamental and technological interest^2^,^3^,^4^,^5^ can emerge when ferroelectricity is influenced by external factors, such as system dimensionality^6^, strain gradients^7^,^8^,^9^, electrostatic coupling^10^,^11^ and magnetic interaction^12^,^13^.

Here we report the spontaneous formation of an extended coherent three-dimensional (3D) superlattice in the nominal ferroelectric phase of specifically grown potassium–lithium–tantalate–niobate (KLTN) crystals^14^,^15^,^16^,^17^. Visible-light propagation reveals a polarization super-crystal with a micrometric lattice constant, a counterintuitive mesoscopic phase that naturally mimics standard solid-state structures but on scales that are thousands of times larger. The phenomenon is achieved using compositionally disordered ferroelectrics^18^,^19^,^20^,^21^,^22^,^23^,^24^,^25^,^26^,^27^. At one given temperature, these have the interesting property of manifesting a single perovskite phase whose dielectric properties depend on the specific composition^28^,^29^,^30^. For example, a compositional gradient along the pull axis leads to a position-dependent Curie point *T*_C_(**r**), so that for a given value of crystal temperature *T* a phase separation occurs, where regions with *T*>*T*_C_ are paraelectric and those with *T*<*T*_C_ have a spontaneous polarization^31^. Specifically tailored growth schemes are even able to achieve an oscillating *T*_C_ along a given direction, say the *x* axis^32^,^33^. Under these conditions, we can expect that, at a given *T* in proximity of the average (macroscopic) *T*_C_, the sample will be in a hybrid state with alternating regions with and without spontaneous polarization. Crossing the Curie point, under conditions in which perovskite polar domains pervade the volume forming 90° configurations to minimize the free energy associated with polarization charge^34^, this oscillation can form a full 3D periodic structure.

## Results

### Observation of a compositionally induced super-crystal

**Figure 1:**
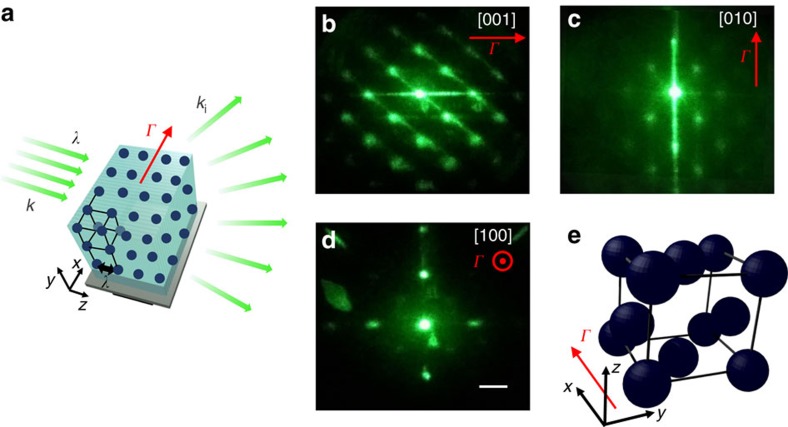
Super-crystal in the ferroelectric phase. (**a**) Sketch of visible-light diffraction from micrometric structures through a transparent crystal and (**b**–**d**) 3D superlattice probed at *T*=*T*_C_−2 K along the principal symmetry direction of the crystal, respectively, with the incident wavevector **k** parallel to (**b**) the *z* direction, (**c**) *y* direction and (**d**) *x* direction. Crystallographic analysis reveals the elementary cubic structure of lattice constant *Λ* shown in **e**. Scale bar, 1.2 cm.

To investigate the matter, we make use of top-seeded ferroelectric crystals with an oscillating composition along the growth axis achieved using an off-centre growth technique in the furnace^33^,^35^. We obtain a zero-cut 2.4 mm by 2.0 mm by 1.7 mm, along the *x*,*y*,*z* directions, respectively, optical-quality KLTN sample with a periodically oscillating niobium composition of period *Λ*=5.5 μm along the *x* axis, with an average composition K_1−*α*_Li_α_Ta_1−*β*_Nb_β_O_3_, where *α*=0.04 and *β*=0.38 (see Methods). When the crystal is allowed to relax at *T*=*T*_C_−2 K, that is, in proximity of the spatially averaged room-temperature Curie point *T*_C_=294 K, laser light propagating through the sample suffers relevant scattering with strongly anisotropic features (Fig. 1a–d). Typical results are reported in Fig. 1b–d, and they appear as an optical analogue of X-ray diffraction in low-temperature solids. This optical diffractometry provides basic evidence of a 3D superlattice at micrometric scales. Probing the principal crystal directions reveals several diffraction orders that map the entire reciprocal space. The large-scale super-crystal, which permeates the whole sample, overlaps—along the *x* direction—with the built-in compositional oscillating seed (see Methods). The superlattice extends in full three dimensions, with the same periodicity *Λ*=5.5 μm of the *x*-oriented compositional oscillation, also along the orthogonal *y* and *z* directions. In particular, Fig. 1d indicates that in the plane perpendicular to the built-in dielectric microstructure Γ vector, that is, where spatial symmetry should be unaffected by the microstructure in composition, the ferroelectric phase transition leads to a spontaneous pattern of transverse scale *Λ*. The corresponding elementary structure on micrometric spatial scales is reported in Fig. 1e; it can be represented as a face-centred cubic structure in which the occupation of one of the three faces (*z*−*y* face) is missing^36^. The structure, which is, to our knowledge, not observed at atomic scales, can be reduced to a simple cubic structure with a threefold basis and lattice parameter *a*=Λ.

**Figure 2:**
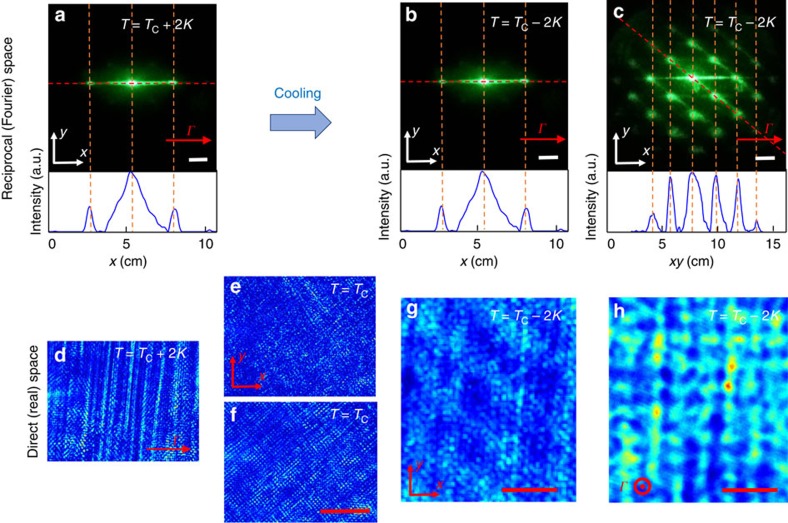
Light diffraction above and below T_c_. (**a**) Reciprocal space probed at *T*=*T*_C_+2 K (hot paraelectric phase), showing the first diffraction orders due to the one-dimensional sinusoidal compositional modulation. Cooling below the critical point results at *T*=*T*_C_−2 K (super-crystal ferroelectric phase) in (**b**) a supercooled (metastable) 1D superlattice with the same diffraction orders that relaxes at the steady state into (**c**) the cold (stable) super-crystals. In both **b**,**c** the direction of incident light is othogonal to Γ, as in **a**. (**d**–**h**) Corresponding transmission microscopy images revealing (**d**) unscattered optical propagation, (**e**,**f**) scattering at the phase transition, (**g**) unscattered optical propagation in the metastable superlattice and (**h**) periodic intensity distribution underlining the 3D superlattice. Metastable and stable (equilibrium) phases are inspected, respectively, at times *t*≈1 min and *t*≈1 h after the structural transition at *T*=*T*_C_. Bottom profiles in **a**–**c** are extracted along the red dotted line. Scale bars (**a**–**c**), 1.2 cm, (**d**–**f**), 100 μm and (**g**,**h**), 10 μm.

As the crystal is brought below the average Curie point, it manifests a metastable (supercooled) and a stable (cold) phase, as analysed in Fig. 2 both in the reciprocal (Fourier) and direct (real) space. In the nominal paraelectric phase, at *T*=*T*_C_+2 K (Fig. 2a), we observe the first Bragg diffraction orders (±1) consistent with the presence of the seed microstructure, a one-dimensional (1D) transverse sinusoidal modulation acting as a diffraction grating; the distance from the central zero order fulfills the Bragg condition, that is, scattered light forms an angle *θ*_B_=*λ*/2*n*_0_Λ≃7° with the incident wavevector **k**. Crossing the ferroelectric phase-transition temperature *T*_C_ (see Methods), we detect a supercooled metastable state that has an apparently analoguous diffraction effect (Fig. 2b) that is dynamically superseded by the stable and coherent cold superlattice phase (Fig. 2c), in which spatial correlations are extended to the whole crystal volume. In real space, transmission microscopy (see Methods) shows unscattered optical propagation through the paraelectric sample at *T*=*T*_C_+2 K (Fig. 2d), which turns into critical opalescence and scattering from oblique random domains at the structural phase transition (Fig. 2e,f), and into unscattered transmission in the metastable ferroelectric phase at *T*=*T*_C_−2 K (Fig. 2g). After dipolar relaxation has taken place, the cold super-crystal appears in this case as a periodic intensity distribution on micrometric scales, as shown in Fig. 2h.

### Spontaneous polarization underlying the ferroelectric superlattice

**Figure 3:**
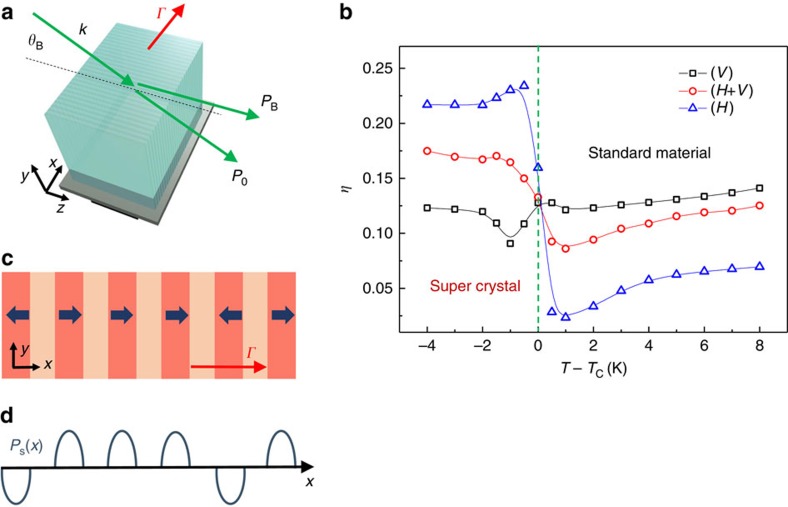
Diffractive behaviour of the 1D supercooled superlattice. (**a**) Sketch of the experimental geometry and (**b**) detected diffraction efficiency (dots) as a function of temperature in the proximity of ferroelectric transition for different wave polarizations. An anomaly appears crossing *T*_C_ for H-polarized light signalling the emergence of the super-crystal. Lines are interpolations serving as guidelines. (**c**) Scheme of the periodically ordered ferroelectric state along the *x* direction undelying the super-crystal phase for *T*<*T*_C_ and giving the spontaneous polarization *P*_S_(*x*) sketched in **d**.

To further analyse these supercooled and cold phases, we inspect the supercooled 1D phase (Fig. 2b) that is accessible through linear (unbiased) and electro-optic (biased) polarization-resolved Bragg diffraction measurements. In particular, referring to the set-up illustrated in Fig. 3a, we measure the diffraction efficiency *η*=*P*_B_/(*P*_B_+*P*_0_), where *P*_B_ and *P*_0_ are, respectively, the diffracted and non-diffracted powers, in the first Bragg resonance condition, that is, with the incident wavevector **k** forming the angle *θ*_B_ with respect to the *z* axis. The diffraction efficiency *η* is reported in Fig. 3b for different input light polarization and temperature across the average Curie point. Diffraction strongly depends both on the nominal crystal phase and on the polarization of the incident wave: a large increase in *η* is found for light polarized in the *x*,*z* plane (H-polarized). For *T*>*T*_C_, the dependence on light polarization is consistent with what expected in standard periodically index-modulated media (wave-coupled theory), that is, a weak temperature dependence and a maximum *η* for light polarized normal to the grating vector (V-polarized). In this case, the difference in *η*_H_ (Δ) and *η*_V_ (□) can be related to the different Fresnel coefficients governing interlayer reflections and is congruently *η*_V_>*η*_H_ by an amount that decreases for larger *θ*_B_ (refs 37, 38). Consistently, the (H+V)-polarized curve (○), that is, when the input linear polarization is at 45° with respect to the H and V polarizations, falls between these two curves. Standard behaviour is violated for *T*<*T*_C_, where a large enhancement in *η*_H_ rapidly leads to a regime with *η*_V_<*η*_H_.

The physical underpinnings of the super-crystal can be grasped considering the simple model illustrated in Fig. 3c. Here we consider the metastable 1D superlattice (Fig. 2b) before tensorial effects cause the full 3D superlattice relaxation (Fig. 2c). Specifically, for a given *T*, regions with a local value of *T*_C_ such that *T*<*T*_C_ (dark shading) will manifest a finite spontaneous polarization *P*_S_≠0, whereas region with *T*>*T*_C_ (light shading) will have a *P*_S_≃0. Optical measurements are sensitive to the square of the crystal polarization 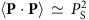 through the resulting index pattern modulated via the quadratic elecro-optic response *δn*(*P*)=−(1/2)*n*^3^*gP*^2^, where *n* is the unperturbed refraction index and *g* is the corresponding perovskite elecro-optic coefficient^25^,^39^. Enhanced Bragg-scattering of light polarized parallel to the seed direction Γ (H in Fig. 3b—super-crystal) indicates that *P*_S_(*x*) is parallel to the seed direction (*x* axis), where the elecro-optic coefficient *g* has its maximum value *g*=0.16 m^4 ^C^−2^. The resonant response at *θ*_B_ and the absence of higher harmonics (Fig. 2b) indicate that this *P*_S_(*x*)^2^ distribution is sinusoidal with wavevector Γ. Hence, although in general it may be that macroscopically 〈**P**〉≃0, it turns out that 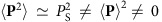 on the micrometric scales, in analogy with optical response in crystals affected by polar nanoregions^25^,^27^,^40^. Optical diffraction efficiency reported in Fig. 3b then occurs considering 
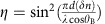
, with resonant enhanced diffraction for *T*<*T*_C_ caused by *δn*=*δn*_0_+*δn*(*P*), where *δn*_0_∼10^−4^ is the polarization-independent index change due to the periodic composition variation (Sellmeier's index change).

### Electro-optical diffraction analysis

**Figure 4:**
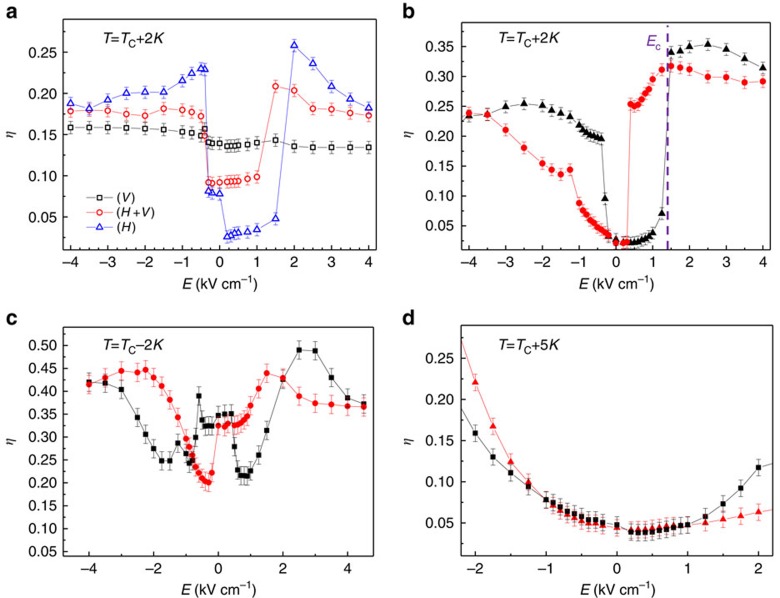
Electro-optic Bragg diffraction in the critical region. (**a**) Diffraction efficiency as a function of the external applied field for different light polarizations at *T*=*T*_C_+2 K; (**b**) hysteresis loop at the same temperature and (**c**) at *T*=*T*_C_−2 K for H-polarization. (**d**) Expected 32 weak-histeretic paraelectric (parabolic) behaviour at *T*=*T*_C_+5 K. In **b**–**d**, black and red dots indicate data obtained, respectively, increasing and decreasing the bias fields. Lines are interpolations serving as guidelines. Error bars are given by the statistics on five experimental realizations.

To validate this picture, we perform electro-optic diffractometry experiments, in which a macroscopic polarization activating the nonlinear periodic response is induced via an external static field *E* applied along *x*. Results are reported in Fig. 4; in particular, in Fig. 4a the polarization and field dependence of *η* are shown at *T*=*T*_C_+2 K. We observe a nearly field-independent behaviour for V-polarized light, which arises from its low electro-optic coupling (bias field and light polarization are orthogonal, *g*=−0.02 m^4 ^C^−2^); differently, *η*_H_ increases with the field showing a ‘discontinuity' at the critical field *E*_C_=(1.4±0.1) kV cm^−1^. The strong similarity between this enhancement and those observed under unbiased conditions at *T*_C_ (Fig. 3b) indicates that *E*_C_ coincides with the coercive field, and the discontinuity corresponds to the field-induced phase transition^16^,^26^,^35^. In fact, in Fig. 4b we repeat this experiment, enhancing the experimental field sensitivity and acquiring data also for decreasing field amplitudes. The result is a partial hysteretic loop for the diffraction efficiency that demontrates the field-induced transition and underlines that, both in the linear and nonlinear (electro-optic) case, the effect of the seeded ferroelectric ordering is to provide a periodic spontaneous polarization along *x*. We also note a slight asymmetry with respect to positive/negative fields; this is associated with a residual fixed space–charge field that may play an important role in the spontaneous polarization alignment process and hence in leading to a residual 〈**P**〉≠0. The existence of a periodic spontaneous polarization distribution in the superlattice (Fig. 3c) is confirmed in Fig. 4c, where electro-optic Bragg diffraction below *T*_C_ is reported. An oscillating full-hysteretic behaviour is observed as a function of the external field, consistently with the prediction 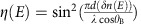 with 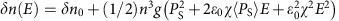. The increase in *η* due to the superlattice polarization allows us to explore its full sinusoidal behaviour, which usually requires extremely large fields in the paraelectric phase and reduces to a parabolic behaviour (Fig. 4d)^41^. From this parabolic behaviour detected at *T*=*T*_C_+5 K we estimate that the resulting ampitude in the point-dependent Curie temperature due to the compositional modulation is Δ*T*_C_≃2 K (ref. 32). Agreement with the periodic polarization model is further stressed by deviations emerging in *η*(*E*), especially for low and negative increasing fields, where the dependence on 〈*P*_S_〉 makes observations weakly dependent on the specific experimental realization.

## Discussion

**Figure 5:**
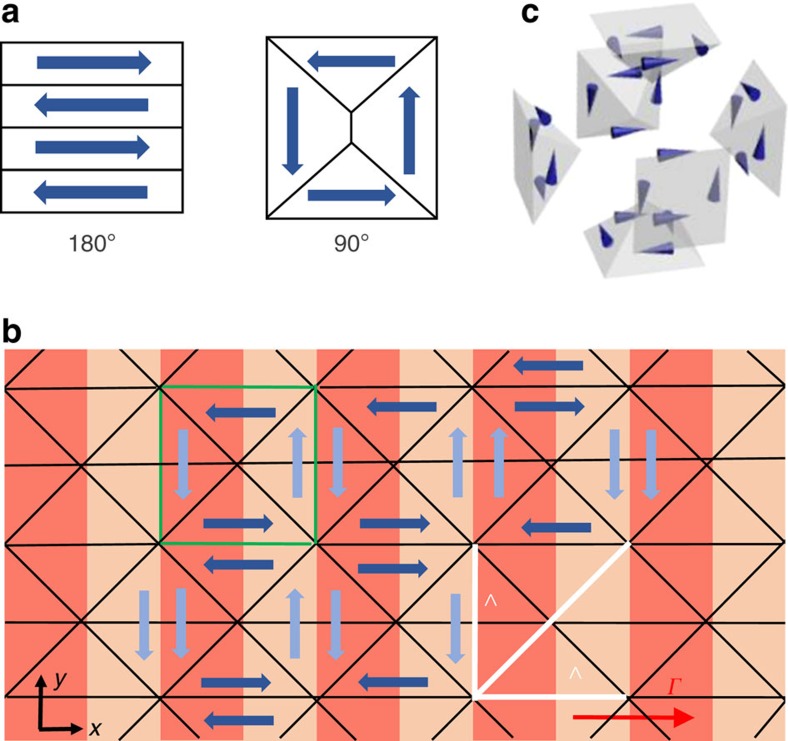
Polar-domain configuration underlying the 3D superlattice. (**a**) Typical 180° and 90° domain configurations in perovskite ferroelectrics. (**b**) Planar domain arrangement scheme in the stable super-crystal phase obtained with elementary blocks of 90° configurations (green cell). In this periodically ordered ferroelectric state, the compositional modulation (as for Fig. 3c), other domain walls ruling optical diffractometry (black lines), and periods along *x*, *y* and *xy* axes (white bars) are highlighted. Vertical polarizations have a lighter colour to stress their weak optical response in our KLTN sample. (**c**) Extension of the single unit cell (green cell in **b**) in three dimensions.

An interesting point arising from the experimental results and analysis is how the periodically ordered polarization state along the *x* direction leads to the super-crystal. Since we pass spontaneously from a metastable to a stable mesoscopic phase, polar-domain dynamics in the presence of the fixed spatial scale *Λ* play a key role. In fact, we note that the 1D superlattice sketched in Fig. 3c involves the appearence of charge density and associated strains between polar planes, so that the ferroelectric crystal naturally tends to relax into a more stable configuration. In standard perovskites, equilibrium configurations are mainly those involving a 180° and 90° orientation between adjacent polar domains, as schematically shown in Fig. 5a. To explain the 3D polar state and its periodical features underlying the super-crystal, we consider the 90° configuration, which is characterized by 45° domain walls that we observe in a disordered configuration during the ferroelectric phase transition at *T*_C_ (Fig. 2f). Owing to the periodic constraint along the *x* axis, this arrangement has the unique property of reproducing our observations, minimizing energy associated to internal charge density and transferring the built-in 1D order to the whole volume with the same spatial scale *Λ*. We illustrate the domain pattern in Fig. 5b for the *x*−*y* plane, whereas in Fig. 5c the elementary cell is shown in the 3D case, where it maintains its stability features in terms of charge density energy. In particular, in Fig. 5b, domain walls resulting in the diffraction orders of Fig. 1b are marked, as well as the 45° correlation period, which agrees with optical observations of the reciprocal space. We further stress that vertical domains (light blue in Fig. 5b) are optically analoguous to paraelectric regions; moreover, 180° rotations in the polarization direction in each polar region has no effect on the optical response. In view of the symmetry of this arrangement, the observed diffraction anisotropy (Fig. 1d) is then associated to the absence of grating planes in the *y*−*z* face.

Further insight on the 3D domain structure requires numerical simulations based on Monte Carlo methods^42^,^43^ and phase-field models^44^,^45^,^46^,^47^; they may confirm our picture and reveal new aspects for ferroelectricity, such as polar dynamics, spontaneous long-range ordering and the role of polar strains in composite ferroelectrics with built-in compositional microstructures. In fact, the effect of the composition profile is here crucial in triggering the spontaneous formation of the macroscopic coherent structure, as it sets the typical domain size along the *x* direction and so rules the whole dynamic towards the equilibrium state. We expect that a different amplitude and period of the modulation may affect the formation, stability, time- and temperature dynamics of the super-crystal; indeed, the parameters of the compositional gradient may be important in determining the interaction between polar regions. Advanced growth techniques^32^ can open future perpectives in this direction, as well as towards composite ferroelectrics with different compositional shapes of fundamental and applicative interest.

To conclude, we have reported the formation of a mesoscopic polarization super-crystal in a nanodisordered sample of KLTN. The large-scale coherent state is triggered by a periodically modulated change in composition. Our results show how ferroelectricity can be arranged into new phases, so that in proximity of an average critical temperature a structural order can emerge with a micrometric lattice constant so as to cause light to suffer diffraction as occurs for X-rays in standard crystals. The effect not only opens new avenues in the optical exploration of critical properties and large-scale structures in disordered systems, but also suggests methods to predict and engineer new states of matter. It can also have an impact on the development of innovative technologies, such as nonvolatile electronic and optical structured memories^2^,^3^,^4^, microstructured piezo devices and spatially resolved miniaturized electro-optic devices^27^,^41^,^48^.

## Methods

### Growth and properties of the microstructured KLTN sample

We consider a compositionally disordered perovskite of KLTN, K_1−*α*_Li_α_Ta_1−*β*_Nb_β_O_3_ with *α*=0.04 and *β*=0.38, grown through the top-seeded solution method by extracting a zero-cut 2.4 mm by 2.0 mm by 1.7 mm, along the *x*, *y*, *z* directions, respectively, optical-quality specimen. It shows, through low-frequency dielectric spectroscopy measurements, the spatial-averaged Curie point, which signals the transition from the high-temperature symmetric paraelectric phase to the low-temperature ferroelectric phase, at the room temperature *T*_C_=294 K. A 1D seed microstructure is embedded into the sample as it is grown through the off-centre growth technique so as to manifest a sinusoidal variation in the low-frequency dielectric constant, and thus in the critical temperature *T*_C_, along the growth axis (*x* direction)^33^,^35^. This dielectric volume microstructure causes an index of refraction oscillation of period *Λ*=5.5 μm, which is able to diffract light linearly and electro-optically^49^. Details on the technique employed in the sample growth can be found in ref. 33. We note here that the composition amplitude of the periodic microstructure can be estimated from Δ*β*/Δ*T*, where Δ*β* is the amplitude variation in niobium composition and Δ*T* is the change in the growth temperature incurred by the off-centre rotation. At the growth temperature of ∼1,470 K, the ratio Δ*β*/Δ*T*≈0.35 mol K^−1^ has been extracted from the phase diagram of KTN. The temperature variation incurred by the off-centre rotation was measured to be 3 K, from which we obtain Δ*β*≈1.05‰ mol.

### Optical diffraction experiments

The macroscopic linear and electro-optic diffractive properties of the crystal have been investigated launching low-power (mW) plane waves at *λ*=532 nm that propagate normally and parallelly to the grating vector **Γ** (Γ=2*π*/*Λ*), which is along the *x* direction (Fig. 3a). Light diffracted by the medium is detected using a broad-area CCD (charge-coupled device) camera placed at *d*=0.2 m from the crystal output facet or collected into Si power meters. In real-space measurements (Fig. 2d–h), the output crystal facet is imaged on the CCD camera and a cross-polarizer set-up^25^,^27^ has been used to highlight contrast due to polarization inhomogeneities. The time needed to obtain a fully correlated state corresponding to the 3D super-crystal depends on the cooling rate *τ* and on the details of the thermal environment. Considering, for instance, as a thermal protocol a cooling rate *τ*=0.05 K s^−1^ and an environment at *T*=*T*_C_+1 K (weak thermal gradients), we have found that the metastable 1D lattice state at *T*=*T*_C_−2 K (Fig. 2b), in which correlations involve mainly in the direction including the Γ vector, lasts ∼1 h. In this stage, although no macroscopic order occurs in the other directions^50^, we observe optimal optical transmission of the sample (Fig. 2g); output light is not affected by scattering related to the existence of random domains and this undelines the presence of a mesoscopic ordering process in which the typical domain size is set. As regards the inspected temperature range, we have found that the super-crystal forms for temperatures till *T*=288 K, although correlations are weaker at the lower temperatures. This is consistent with the fact that at these temperatures also the regions with a lower local *T*_C_ are well below the transition point.

## Additional information

**How to cite this article:** Pierangeli, D. *et al*. Super-crystals in composite ferroelectrics. *Nat. Commun.* 7:10674 doi: 10.1038/ncomms10674 (2016).
